# 

**Published:** 1856-06

**Authors:** 


### RECEIPTS ON SUBSCRIPTIONS,

FROM APRIL 16TH TO MAY 20TH, 1856.

**ILLINOIS.**

| | | |
|---|---|---|
| Dr. C. J. French, | Vol. 5, | $2 00 |
| " J. T. Pearman, | " 5, | 2 00 |
| " A. Patterson, | " 4, | 2 00 |
| " John Allen, | " 5, | 2 00 |
| " E. C. Banks, | Vol. 4 & 5, | 5 00 |
| " R. Rouse, | Vol. 5, | 2 00 |
| " V. R. Bridges, | " 5, | 2 00 |
| " T. D. Fitch, | " 5, | 2 00 |
| " Mills & Herrick, | " 5, | 2 00 |
| " A. M. Johnson, | Vol. 3 & 4, | 5 00 |

**INDIANA.**

| | | |
|---|---|---|
| Dr. Wm. Townsend, | Vol. 5, | $2 00 |
| " C. C. Cornett, | " 5, | 2 00 |
| " J. H. Spurrier, | Vol. 4 & 5, | 4 00 |

**INDIANA—Continued.**

| | | |
|---|---|---|
| Dr. Sam'l Duggy, | Vol. 5, | 1 50 |
| " Wm. T. Green, | " 5, | 2 00 |

**MICHIGAN.**

| | | |
|---|---|---|
| Dr. N. A. Hill, | Vol. 5, | $1 00 |
| " Alex. DeArmond, | " 5, | 2 00 |
| " H. Knapp, | " 5, | 2 00 |

**WISCONSIN.**

| | | |
|---|---|---|
| Dr. N. T. Millard, | Vol. 5, | $2 00 |
| " J. O. Beals, | Vol. 4 & 5, | 5 00 |

**IOWA.**

| | | |
|---|---|---|
| Dr. J. Williamson, | Vol. 4, | $1 00 |

